# Gunshot Wound of the Abdomen. A Case

**Published:** 1890-09

**Authors:** Sam Cunningham

**Affiliations:** Elgin


For Daniel's Texas Medical Journal.

### 'A CASE OF GUNSHOT WOUND OF THE ABDOMEN."

BY SAM CUNNINGHAM, M. D., ELGIN.

Read at Meeting Austin District Medical Socieiy, and Referred to Publishing Committee for Publication.

ON THE 17th day of April last Tom McLemore, white, age 18 years, received an accidental shot from a target gun, the ball entering the abdomen two inches below and an inch to the right of the umbilicus, and having an upward range going through, its point of exit being in the back just above the right kidney.

Dr. C. C. Rutherford, of McDade, was immediately called, who seeing the conditions had only to wait for further developments, giving only the necessary amount of opiates to relieve pain and allay nervous shocks. Patient had on third day after the accident natural passages from both bowel and kidneys, showing no appreciable signs of mischief.

Dr. R. thinking the patient would get along by the exercise of proper precaution, discontinued his attentions, instructing the family to notify him should any harm accrue later. On the morning of the sixth day the patient from the exercise of imprudence, both in too much eating, and physical exercise, was seized with violent pains through the bowels, some swellings and exceeding tenderness over the region of the transverse colon. The bowel was constipated, though there was not much tympanites. Dr. R. was again called. Although a general line of treatment was pursued, the patient continued to grow worse; when on the ninth day of the accident I was requested to go in consultation. The peritonitis at this time was well marked, the bowel distended, his countenance anxious, skin cold, and clammy. The history of the case for the prior 24 hours to my visit, was occasional exacerbations of the temperature, with pain more or less prominent all the time. The pain was now so intense that we gave hypodemically a dose of morphine and atropia, combining also digitalis, as the pulse was rather feeble and compressible. Vomiting soon became a prominent and persistent symptom. At intervals of about every six hours vomiting of fecal matter would ensue. This condition of affairs kept up continuously for eight days, and his whole appearance was one of progressive prostration. The treatment employed in this case was a general one, viz: Opiates sufficient to promote ease, hot turpentine stupes vigorously applied to the bowels, whiskey and all the liquid nourishment that could be given ; sweet oil, too, was given with the hope of overcoming or rather disengaging the fecal impaction. The oil was given shortly after the vomiting spells, as the stomach would appear more tolerant then. Enemas were also administered occasionally. On the 17th day after the occurrence, when after one of the many attempts to prompt the action of bowel by an enema, the patient had a large action of the most foul kind and odor, containing some hard lumps, which confirmed the diagnosis of "impaction," or possibly "intussusception." The patient after this ceased to vomit; symptoms got better at once, and he made a rapid recovery.

I do not report this case claiming the merit of any special management, but hoped that it might prove of some interest to the Society.

